# Time to initiation of antenatal care and its predictors among pregnant women who delivered in Arba Minch town public health facilities, Gamo Zone, southern Ethiopia, 2023: a retrospective follow-up study

**DOI:** 10.1186/s12978-024-01818-w

**Published:** 2024-05-31

**Authors:** Abebe Gedefaw Belete, Mesfin Kote Debere, Mekdes Kondale Gurara, Negusie Boti Sidamo, Mulugeta Shegaze Shimbre, Manaye Yihune Teshale

**Affiliations:** 1 School of Public Health, College of Medicine and Health Sciences, Arba Minch University, Arba Minch, Ethiopia; 2 School of Nursing and Public Health, Public Health Medicine Discipline, University of KwaZulu-Natal, Durban, South Africa; 3 Department of Health Promotion, CAPHRI Care and Public Health Research Institute, Maastricht University, Maastricht, Netherlands

**Keywords:** Antenatal care, Time to initiation, Public health facilities, Southern Ethiopia

## Abstract

**Background:**

Early antenatal care visit is important for optimal care and health outcomes for women and children. In the study area, there is a lack of information about the time to initiation of antenatal care. So, this study aimed to determine the time to initiation of antenatal care visits and its predictors among pregnant women who delivered in Arba Minch town public health facilities.

**Methods:**

An institution-based retrospective follow-up study was performed among 432 women. A systematic random sampling technique was employed to select the study participants. The Kaplan-Meier survival curve was used to estimate the survival time. A Multivariable Cox proportional hazard regression model was fitted to identify predictors of the time to initiation of antenatal care. An adjusted hazard ratio with a 95% confidence interval was used to assess statistical significance.

**Results:**

The median survival time to antenatal care initiation was 18 weeks (95% CI = (17, 19)). Urban residence (AHR = 2.67; 95% CI = 1.52, 4.71), Tertiary and above level of education of the women (AHR = 1.90; 95% CI = 1.28, 2.81), having pregnancy-related complications in a previous pregnancy (AHR = 1.53; 95% CI = 1.08, 2.16), not having antenatal care for previous pregnancy (AHR = 0.39; 95% CI = 0.21, 0.71) and unplanned pregnancy (AHR = 0.66; 95% CI = 0.48, 0.91) were statistically significant predictors.

**Conclusion:**

Half of the women initiate their antenatal care visit after 18 weeks of their pregnancy which is not in line with the recommendation of the World Health Organization. Urban residence, tertiary and above level of education of the women, having pregnancy-related complications in a previous pregnancy, not having previous antenatal care visits and unplanned pregnancy were predictors of the time to initiation of antenatal care. Therefore, targeted community outreach programs including educational campaigns regarding antenatal care for women who live in rural areas, who are less educated, and who have no previous antenatal care experience should be provided, and comprehensive family planning services to prevent unplanned pregnancy are needed.

## Background

Antenatal care (ANC) is the care provided by skilled health professionals to pregnant women to ensure the best health conditions for both the mother and the baby during and after pregnancy. Risk identification, prevention and management of pregnancy-related complications, health education and promotion are the major components of ANC [1]. ANC reduces maternal and newborn morbidity and mortality both directly, through the identification and treatment of pregnancy-related complications and indirectly, through the detection of women at high risk of developing complications during labour and delivery, thus enabling referral to an appropriate level of care. In 2016, the WHO recommended a minimum of eight ANC contacts, with the first contact to take place in the first trimester [2].

Every two minutes, somewhere on our planet especially in low-income countries, women of reproductive age die from problems linked to pregnancy and childbirth [3]. According to WHO, in 2020, there were 287,000 maternal deaths, 2.4 million neonatal deaths and an estimated 2 million stillbirths, almost all of which occurred in low and middle-income countries, especially in sub-Saharan Africa and South Asia [3–6]. By 2030, one of the targets of ending preventable maternal and neonatal mortality is to lower the global maternal mortality ratio (MMR) to less than 70 per 100,000 live births and to reduce neonatal mortality to at least as low as 12 per 1,000 live births [7]. Early ANC initiation plays an important role in achieving the above goals. It maximizes the probability of adherence to the clinically indicated check-up schedule while giving healthcare providers enough time to identify and successfully treat diseases like syphilis, anaemia, malaria, and hypertension and to alleviate and mitigate health conditions that may lead to complications for the mother and baby later on [8].

However globally, the prevalence of early ANC bookings was only 58.6%. In developing countries, only half of the women initiated ANC within the first trimester of pregnancy. ANC within the first trimester of pregnancy was 69.1% and 68.1%, in Asia, and Latin America respectively and 40.8% in Sub-Saharan Africa (SSA) [9]. Although there has been good progress in the coverage of early antenatal care visits; it is still low in developing countries, especially in SSA [10]. In Ethiopia, only 28% of the woman had their first ANC contact during the first trimester [11].

According to previous studies, socio-demographic factors such as maternal age and wealth status, and obstetrics factors such as previous ANC use were found to be important determinant factors for the time of ANC visit initiation [8, 12–15]. In Ethiopia, particularly in the study area, studies targeted the time of antenatal care initiation (survival time) was scarce and they were limited to few variables. Therefore, this study aimed to assess the time to initiation of antenatal care visit and its predictors among pregnant women who delivered in Arba Minch town public health facilities and the finding will provide important evidence on time to initiation of antenatal care visit to concerned bodies to take evidence-based intervention according to the identified predictors. Thus, it will be useful in reducing maternal and neonatal morbidity and mortality by promoting early initiation of antenatal care visits.

## Methods

### Study setting, design and period

An institution-based retrospective follow-up study was carried out at public health facilities in Arba Minch town. Arba Minch is located in the Gamo Zone, southern Ethiopia approximately 500 km south of Addis Ababa and 275 km from Hawassa at an elevation of 1285 m above sea level. It has one general and one primary public hospital with two health centres:- Naming Arba Minch General and Dilfana primary hospitals, Secha and Woze health centers. There were a total of 953 healthcare providers in all health facilities. All facilities provide maternal health services like ANC, delivery and post-natal care, in addition to other routine health services. The ANC and delivery services were mainly given by midwives [16]. According to the “Population Projection of Towns as of July 2021” conducted by the CSA ( SCentral Statistical Agency), this town has a total population of 192,043 [17]. The study was conducted from June 01, 2023, to June 30, 2023.

### Population

All pregnant women who delivered in Arba Minch town public health facilities were the source population whereas all selected pregnant women who delivered in Arba Minch town public health facilities during the study period who fulfilled the inclusion criteria were the study population. Women who were unable to respond due to severe illness or whose gestational age during antenatal care initiation was not registered in their ANC follow-up cards were excluded.

### Sample size determination and sampling technique

The sample size was determined by using Stata17.0 by taking a confidence level of 95%, power of 80%, and a 10% withdrawal. Having a higher education level, being an urban resident, and being the richest were found to be major predictor variables from a previous study and the final sample size was 436 [12, 14]. The study was done in one month and the average number of deliveries per month in all health facilities was 1302. So, the systematic random sampling technique with a K value of 3 (1302/436) was used to get the respective respondents from each health facility. We collected our data during delivery so as to get those mothers who came for delivery without having ANC throughout pregnancy (censored mothers).

### Variables

The time to initiation of antenatal care visit was the outcome variable whereas Socio-demographic and socio-economic factors like residence, age, marital status, educational status, occupation, religion, average monthly income, family size, partner education and occupation, obstetric factors like age at marriage, gravidity, parity, birth interval, age at first birth, place of delivery for previous pregnancy, mode of delivery for previous pregnancy, previous ANC use, previous pregnancy complication, pregnancy intention, means of pregnancy recognition, autonomy on a decision, and partner involvement during ANC were the independent variables.

### Operational definitions

#### Time to initiation of ANC

The time was measured in weeks from the date of pregnancy to the time of ANC initiation [18].

#### Event

The event was considered to occur if the pregnant woman had at least one ANC visit throughout pregnancy.

#### Censored

The event was considered censored if a pregnant woman delivered without any ANC visit.

#### Pregnancy-related complications

Are defined as health problems that occur during Pregnancy and affect the mother’s health, baby’s health or both [19].

### Data collection and management

A structured interviewer-administered questionnaire was prepared after reviewing different literature to collect relevant data [8, 12–15]. The data were collected by two health professionals with one supervisor. Data collectors obtained all relevant data related to socio-demographic, socio-economic, and obstetric-related factors by interviewing a woman and the outcome variable by reviewing ANC cards. The data collection tool was first prepared in English language and translated into Amharic language and then back to English. A 5% pretest was performed. Two days of training were provided to data collectors. The supervision was performed at each step of data collection and the collected data were checked for consistency and completeness before data analysis.

### Data processing and analysis

Data collection was performed using Kobo collect and Stata 17.0 was used for data cleaning and statistical analysis. Descriptive analysis, such as the mean with standard deviation, median with interquartile range, frequency and percentages were used to describe different variables. A Kaplan-Meier (KM) survival curve was used to estimate the median survival time and a Log-rank test was used to compare the survival probabilities. Cox proportional hazard regression analysis was used to identify predictor variables. Independent variables having a *P*-value ≤ 0.25 in the bivariable analysis were fitted into the final multivariable model for further analysis. Multi-collinearity between variables was checked using a variance inflation factor with a cut-off point with a median VIF < 5. The assumptions of the Cox proportional hazard regression model were checked based on the Schoenfeld residual global test and the goodness fit of the model was checked by using the Cox–Snell residual and Nelson Aalen cumulative hazard plot. Adjusted hazard ratios (AHRs) with 95% confidence intervals (CIs) and *P*-value ≤ 0.05 were used to assess the strength of association and statistical significance.

## Results

### Socio-demographic characteristics

Out of 436 women who were invited to participate, (99%) were interviewed and included in the analysis. The mean age of the women was 26.26 years, with a standard deviation of (± 4.78) years. The majority of the women (93.7%) were urban dwellers (Table 1).

**Table 1:** Socio-demographic characteristics of pregnant women who delivered in Arba Minch town public health facilities, Gamo zone, Southern Ethiopia, 2023 (*n* = 432)

Variable	Categories	Frequency	Percentage
Age of the mother	15–19	34	7.9
	20–24	110	25.4
	25–29	161	37.3
	30–34	70	16.2
	35–45	57	13.2
Residence	Urban	405	93.7
	Rural	27	6.3
Family Size	≤ 2	162	37.5
	3–4	225	52.0
	≥ 5	45	10.5
Religion	Muslim	41	9.5
	Orthodox	160	37.0
	Protestant	231	53.5
Average Monthly Income	< 5,000	93	21.5
	5,000–10,000	170	39.4
	> 10,000	169	39.1
Level of Education of the women	Primary and Under-Primary	80	18.5
	Secondary	118	27.3
	Tertiary and Above	234	54.2
Level of Education of the Partner	Primary and Under-Primary	93	21.5
	Secondary	119	27.6
	Tertiary and Above	220	50.9
Occupation of the women	House Wife	184	42.6
	Gov.t employ	118	27.3
	Merchant	73	16.9
	Farmer	57	13.2
Occupation of the Partner	Gov.t employ	174	40.3
	Merchant	157	36.3
	Farmer	75	17.4
	Other(student, driver, NGO)	26	6.0

### Obstetrics and other related factors

Among the study participants, 93% of the women had a history of ANC visits for their previous pregnancy. The majority (83.1%) of the current pregnancies were planned and 16.4% of the women had a previous history of pregnancy-related complications (Table 2).

**Table 2:** Obstetrics and other related characteristics of pregnant women who delivered in Arba Minch town public health facilities, Gamo zone, Southern Ethiopia, 2023

Variable	Categories	Frequency	Percentage
Age at first Marriage	< 18 Years	72	16.7
(*n* = 432)	≥ 18 Years	360	83.3
Gravidity	Primigravida	155	35.9
	Multigravida	277	64.1
Parity	Nulliparity	157	36.3
	Primiparity	129	29.9
	Multi Parity	146	33.8
Birth Interval (*n* = 275)	< 33 month	59	21.4
	≥ 33 month	216	78.6
Age at First Birth	< 18 years	24	8.7
	≥ 18 years	251	91.3
Pregnancy-Related complications	Yes	45	16.4
	No	230	83.6
Place of Delivery for your Last Pregnancy	Home	11	4.0
	Health Facility	264	96.0
Mode of Delivery for Your Last Pregnancy	Vaginal Delivery	234	85.1
	Cesarean Section	41	14.9
ANC Follow-up for Your Last Pregnancy	Yes	257	92.8
	No	20	7.2
ANC for Current Pregnancy (*n* = 432)	Yes	423	97.9
	No	9	2.1
Means of Pregnancy Recognition	Missed Period	279	64.6
	Urine Test	153	35.4
Pregnancy intention	Planned	359	83.1
	Unplanned	73	16.9
Autonomy of Decision on health care utilization	By Myself	53	12.3
	By Partner	13	3.0
	Joint Decision	366	84.7
Partner Involvement during ANC	Yes	343	79.4
	No	89	20.6

### Time to initiation of the first ANC visit

**Fig. 1:**
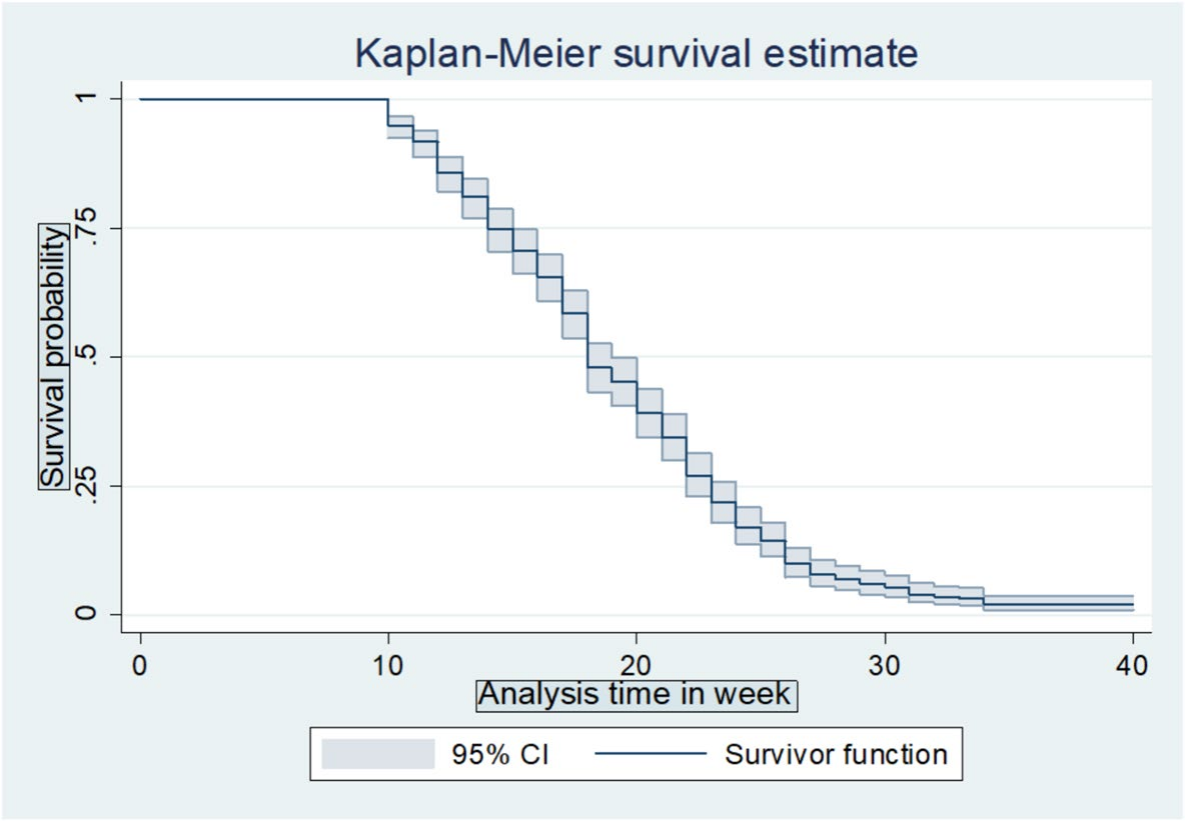
Overall Kaplan-Meier survival curve of the time to initiation of ANC among pregnant women who delivered in Arba Minch town public health facilities, Gamo zone, Southern Ethiopia, 2023

The survival function was computed using the Kaplan-Meier survival estimate. The overall median survival time to the first ANC initiation was 18 weeks (CI = 17, 19). The total follow-up time contributed by all study participants was 8332-person weeks. The cumulative probability of the first ANC initiation was 14.3% at 12 weeks, 52% at 18 weeks, 82.9% at 24 weeks, 94.7% at 30 weeks, and 97.9% at 40 weeks (Fig. 1).

### Comparison of survival probabilities among categories of different independent variables

**Fig. 2:**
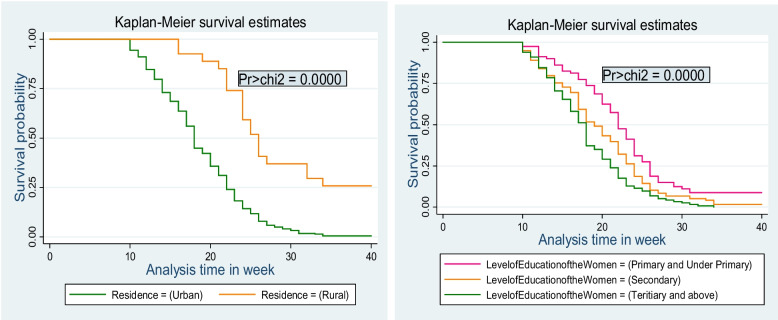
Kaplan-Meier survival curve with a long rank test showing differences in survival probability of the time to initiation of ANC by residence, and level of education among pregnant women who delivered in Arba Minch town public health facilities, Gamo zone, southern Ethiopia, 2023

Kaplan-Meier survival curves together with the log-rank test were performed to compare the survival probabilities among categories of different predictor variables. Women who lived in urban areas had significantly lower survival probability than those who lived in rural areas (Pr > chi2 = 0.0000). Mothers with tertiary and above educational levels had statistically significantly lower survival probability compared to those with secondary and primary level education (Pr > chi2 = 0.0000) (Fig. 2).

### Predictors of time to initiation of ANC visit

#### Bivariable and multivariable Cox regression analysis

In the Bivariable Cox proportional hazard regression analysis, statistical significance was observed among the variables place of residence, average monthly income, level of education of the women, level of education of the partner, birth interval, pregnancy-related complications, ANC follow-up for last pregnancy, and intention of pregnancy (Table 3).

Predictors that had an association at *p*-value ≤ 0.25 in bivariable Cox proportional hazard regression were included in multivariable regression to control the effect of confounders. Finally, place of residence, level of education of the women, pregnancy-related complications, ANC follow-up for the last pregnancy and intention of pregnancy were found to be predictors of the time to initiation of the first ANC visit.

The timing of ANC initiation was 2.67 times shorter among women who lived in urban areas than among those who lived in rural areas (AHR = 2.67; 95% CI = 1.52, 4.71; *P* < 0.001). The timing of ANC initiation was 1.9 times shorter for women who attained a tertiary and above level of education than for those who were in primary and under the primary level of education (AHR = 1.90; 95% CI = 1.28, 2.81; *P* < 0.001). The timing of ANC initiation was 1.53 times shorter for women who had pregnancy-related complications in their previous pregnancy than for those who had no history of pregnancy-related complications in their previous pregnancy (AHR = 1.53; 95% CI = 1.08, 2.16; *P* < 0.015). The timing of ANC initiation among mothers who had no ANC follow-up for their previous pregnancies was longer by 61% as compared to those who had ANC follow-up for their last pregnancy (AHR = 0.39; 95% CI = 0.21, 0.71; *P* < 0.002). The timing of ANC initiation among mothers who had unplanned pregnancies was longer by 34% as compared to those who had planned pregnancies (AHR = 0.66; 95% CI = 0.48, 0.91; *P* < 0.013) (Table 3).

**Table 3:** Bivariable and multivariable Cox proportional hazard regression analysis for predictors of time to initiation of ANC among pregnant women who delivered in Arba Minch town public health facilities, Gamo zone, southern Ethiopia, 2023

Variable	Survival status		Crude hazard ratio (CHR) 95% CI	*P*-value	Adjusted hazard ratio (AHR), 95% CI	*P*-value
	Event	Censored				
	No (%)	No				
**Residence**						
Urban	403 (93.3)	2	3.50 (2.21, 5.54)	0.000*	2.67 (1.52, 4.71)	0.001**
Rural	20 (4.6)	7	1		1	
**Average Monthly Income**						
< 5,000	89 (20.6)	4	1		1	
5,000–10,000	167 (38.7)	3	1.21 (0.94,1.57 )	0.136*	1.05 (0.73, 1.52)	0.759
> 10,000	167 (38.7)	2	1.40 (1.08, 1.81)	0.010*	0.93 (0.63, 1.39)	0.749
**Level of Education of the Woman**						
Primary and Under-Primary	73 (16.9)	7	1		1	
Secondary	116 (26.9)	2	1.48 (1.10, 1.98)	0.009*	1.42 (0.96, 2.11)	0.076
Tertiary and Above	234 (54.2)	0	1.99 (1.52, 2.59)	0.000*	1.90 (1.28, 2.81)	0.001**
**Level of Education of the Partner**						
Primary and Under-Primary	86 (19.9)	7	1		1	
Secondary	118 (27.3)	1	1.44 (1.09, 1.91)	0.010*	0.94 (0.62, 1.41)	0.773
Tertiary and Above	219 (50.7)	1	1.63 (1.26, 2.09)	0.000*	0.86 (0.58, 1.26)	0.445
**Birth Interval**						
< 33 Month	58 (21.1)	1	1		1	
≥ 33 Month	210 (76.4)	6	1.22 (0.91, 1.64)	0.169*	1.15 (0.84, 1.58)	0.357
**Pregnancy-Related Health Problems**						
Yes	45 (16.4)	0	1.93 (1.39, 2.67)	0.000*	1.53 (1.08, 2.16)	0.015**
No	223 (81.1)	7	1		1	
**ANC for Last Pregnancy**						
Yes	256 (92.4)	1	1		1	
No	14 (5.1)	6	0.26 (0.15, 0.46)	0.000*	0.39 (0.21, 0.71)	0.002**
**Pregnancy intention**						
Planned	357 (82.6)	2	1		1	
Unplanned	66 (15.3)	7	0.48 (0.37, 0.63)	0.000*	0.66 (0.48, 0.91)	0.013**

1= reference, * statistically significant at *P*-value ≤ 0.25, ** statistically significant at *P*-value ≤ 0.05

## Discussion

The main goal of the study was to determine the time to initiation of ANC visit and its predictors among pregnant women who delivered in Arba Minch town public health facilities, Gamo zone, Southern Ethiopia, 2023. The overall median time to ANC initiation was 18 weeks. Being an urban resident, having a tertiary and above level of education, having pregnancy-related complications in a previous pregnancy, not having previous antenatal care visit and unplanned pregnancy were found to be the predictors for the time to initiation of antenatal care visit.

According to this study, the median time to ANC initiation was 18 weeks, meaning half of the pregnant women start their ANC visit after 18 weeks of their pregnancy, which is not in line with the 2016 WHO ANC recommendation which is before 12 weeks; this, in turn, implies that many women are at risk of several obstetric complications which may lead to maternal and neonatal morbidity and mortality. This finding is consistent with a study from low and middle-income countries (Malawi, Zambia, Uganda, Yemen, and Mali) [20] and Afghanistan [21] which is 4 months. However, the median time to ANC initiation was earlier as compared to the study performed in Nigeria [15, 22] which is 6 months, which may be due to differences in the study setting: the participants from this study were mainly from an urban area where women had access to education, media and health facilities whereas the study in Nigeria was from the Nigerian demographic and health survey where the majority of the women were from rural areas [15, 22]. The median time to ANC initiation was later than the findings from Paraguay, Indonesia and Cambodia [20]. This variation might be due to socio-demographic and, socio-economic differences.

In this study, place of residence was a significant predictor for time to initiation of ANC visit. Women who reside in urban areas initiate ANC visits earlier than those who live in rural areas. It is in line with a study conducted in Ethiopia [23, 24], Nepal [25], Nigeria [15, 22, 26], and Bangladesh [27]. Similarly, a study performed in low- and middle-income countries stated that women who initiated ANC early were mainly from urban areas [20]. Women from urban areas may be aware of the importance of early ANC initiation and its role in ensuring maternal and child health as they have better educational opportunities [28] and easy access to various media outlets [29]. Additionally, the geographical location of urban areas offers a distinct advantage in terms of healthcare accessibility. Urban regions are typically well-equipped with a variety of healthcare facilities, including hospitals, clinics, maternity centres, and specialized healthcare providers. The proximity of these facilities to residential areas may reduce travel time and logistical challenges, making it easier and more convenient for urban women to access ANC services timely [30, 31].

According to the findings of this study, the level of education of the women was found to be a significant predictor for the time to initiation of ANC visits. Women who have a tertiary and above level of education initiate their ANC visit earlier than those with secondary and primary levels, which is consistent with a study conducted in Ethiopia [8, 12, 14, 18], Nepal [25], Nigeria [15, 22, 26], and Bangladesh [27]. Education enhances women’s capabilities, particularly in understanding and accessing healthcare services [32]. Women with higher educational levels tend to initiate ANC earlier as they have easy access to health-related information and can easily comprehend information conveyed in various formats and methods [24]. Furthermore, educated women are empowered to make independent health decisions, allowing them to take charge of their own healthcare choices, including when and how to initiate ANC, based on their informed understanding and knowledge [32, 33].

Similarly, pregnancy-related complications were found to be another predictor for the time to initiation of ANC visits. Women who faced pregnancy-related complications in their previous pregnancy were more likely to initiate their ANC visit earlier than their counterparts, which is in line with the findings from Ethiopia [23, 34], Rwanda [35] and Tanzania [36]. Women who have previously experienced pregnancy complications or adverse outcomes are more proactive in seeking early ANC services for subsequent pregnancies [37]. Their past experiences may alert them to prioritize comprehensive ANC to prevent similar complications from recurring and to ensure better pregnancy outcomes. Having encountered challenges during a previous pregnancy, these women are often more informed and aware of the potential risks and complications associated with pregnancy. This heightened awareness can motivate them to be more diligent and attentive towards their antenatal health, prompting them to seek timely medical advice and interventions through ANC services [38]. ANC visits in previous pregnancies were found to be another predictor for the time to initiation of ANC visits. Women who had not had ANC service in their previous pregnancy initiated their ANC visit later than those who had. This is in line with the studies from Ethiopia [39, 40], and Afghanistan [21]. Health facilities often serve as crucial platforms for health education and promotion. Women attending ANC previously could receive information, guidance, and counselling on various aspects of pregnancy, ANC, and childbirth. These educational sessions empower them with knowledge related to maternal and child health thus enabling the timely initiation of ANC visits [41]. Women who have no previous ANC experience may not know the services given during ANC, so, they may fear and feel unfamiliar with getting ANC service in health facilities timely [39].

Furthermore, the intention of pregnancy is found to be a predictor for the time to initiation of ANC visits. Women who had unplanned pregnancies were found to initiate ANC visits later than those who had planned pregnancies, which is in line with the studies performed in Nepal [25], Afghanistan [21], Gambia [42], South Africa [43] and south and southwest Ethiopia [13, 44]. Women who find themselves with unplanned pregnancy may experience a range of emotions, including anxiety, sadness or regret. This emotional turmoil can affect their acceptance and attitude towards the pregnancy and can result in late ANC initiation [45]. Additionally, the unexpected nature of unplanned pregnancies can result in delayed recognition and awareness of their pregnancy status and can lead to late ANC initiation [46]. Furthermore, If the partner is not in favour of the pregnancy, he may exert control or dominance over the woman’s healthcare decisions, refusing to grant permission or accompany her to ANC appointments, further restricting her access to essential ANC services [47].

### Limitations of the study

Knowledge and attitude about ANC were not measured due to the retrospective nature of the study. The outcome variable for different women was measured differently as there are different methods for gestational age measurement like; ultrasound measurement, and gestational age based on the last normal menstrual period.

## Conclusion

The median time to ANC initiation was 18 weeks. Half of the pregnant women start their ANC visit after 4.2 months, which is not in line with the 2016 WHO recommendation as it schedules the first contact to take place before three months of pregnancy. Being an urban resident, having a tertiary and above level of education of the women, and having pregnancy-related complications in a previous pregnancy were found to be a positive predictor for antenatal care initiation whereas not having a previous antenatal care visit and having unplanned pregnancy were negative predictors for the time to initiation of antenatal care. Therefore, targeted community outreach programs including educational campaigns regarding ANC for women who live in rural areas, who are less educated, and who have no previous antenatal care experience should be provided, and comprehensive family planning services to prevent unplanned pregnancy are needed.

## Data Availability

The datasets used during the current study are available from the corresponding author upon reasonable request.

## Abbreviations

ANC: Antenatal care
AHR: Adjusted hazard ratio
CHR: Crude hazard ratio
CI: Confidence interval
EDHS: Ethiopian demographic and health survey
KM: Kaplan-meier
NGO: Non-governmental organization
SSA: Sub saharan africa
WHO: World health organization
